# Supplementary material for: spinDrop: a droplet microfluidic platform to maximise single-cell sequencing information content
Source: Nat Commun. 2023 Aug 8;14:4788. doi: 10.1038/s41467-023-40322-w (PMC10409775; doi:10.1038/s41467-023-40322-w)
Supplement: Supplementary file 1 — Supplementary Information [file 41467_2023_40322_MOESM1_ESM.pdf]

spinDrop: a droplet microfluidic platform to maximise single-cell sequencing information content

Joachim De Jonghe[1,2,10], Tomasz S. Kaminski[1,3,10], David B. Morse[4], Marcin Tabaka[5,6], Anna L. Ellermann[1], Timo N. Kohler[1], Gianluca Amadei[7], Charlotte Handford[7], Gregory M. Findlay[2], Magdalena Zernicka-Goetz[7,8], Sarah A. Teichmann[9] & Florian Hollfelder[1,11]

1. Department of Biochemistry, University of Cambridge, Cambridge, United Kingdom
2. Francis Crick Institute, London, United Kingdom
3. Department of Molecular Biology, Institute of Biochemistry, Faculty of Biology, University of Warsaw, Warsaw, Poland
4. Department of Chemistry, University of Cambridge, Cambridge, United Kingdom
5. International Centre for Translational Eye Research, Warsaw, Poland
6. Institute of Physical Chemistry, Polish Academy of Sciences, Warsaw, Poland
7. Department of Physiology, Development and Neuroscience, University of Cambridge, Cambridge, United Kingdom
8. California Institute of Technology, Division of Biology and Biological Engineering, Pasadena, USA
9. Wellcome Trust Sanger Institute, Wellcome Genome Campus, Hinxton, United Kingdom
10. These authors contributed equally
11. Corresponding author, address correspondence to fh111@cam.ac.uk

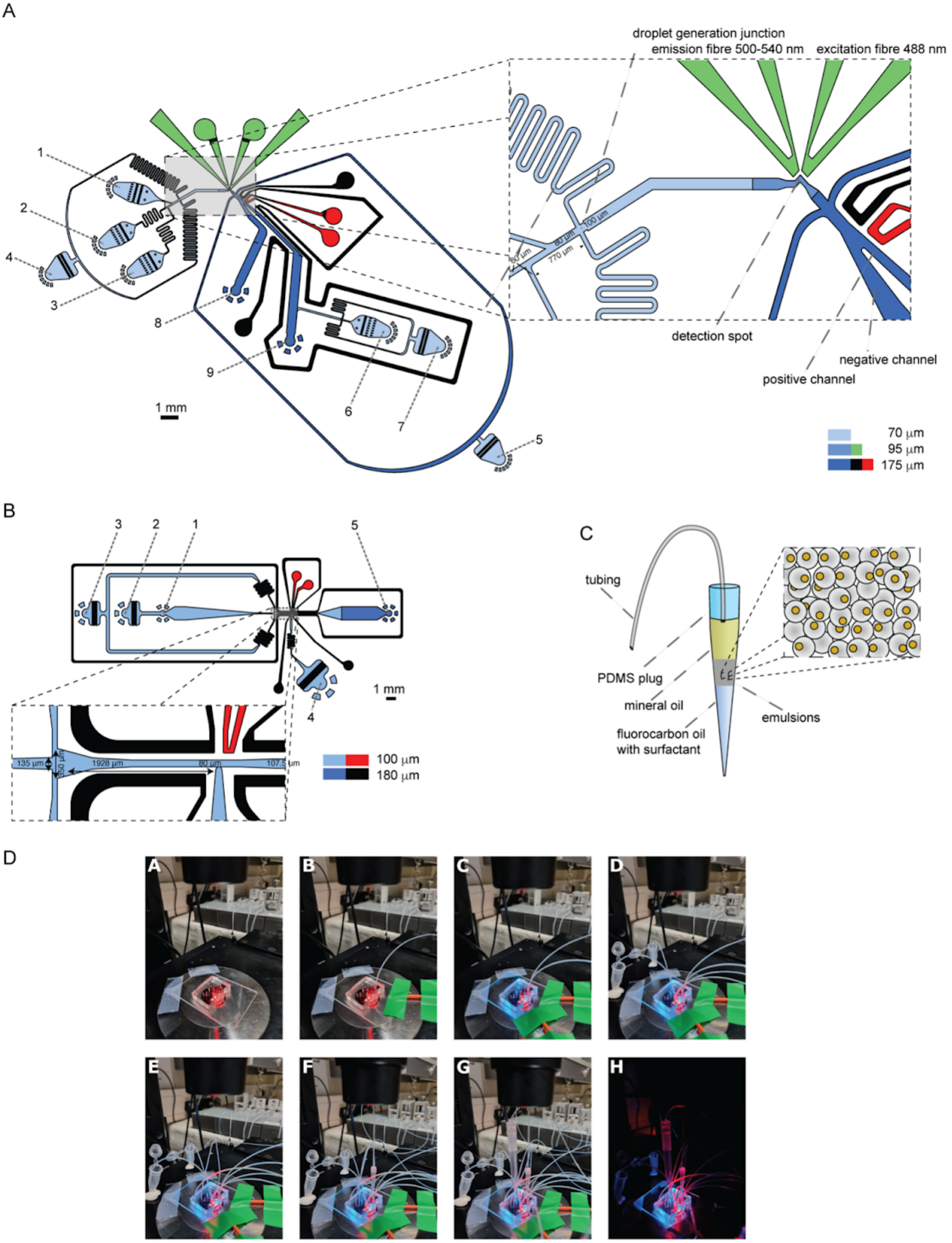

A
droplet generation junction
emission fibre 500-540 nm
excitation fibre 488 nm
1
2
3
4
5
6
7
8
9
1 mm
detection spot
negative channel
positive channel
70 µm
95 µm
175 µm
B
1
2
3
4
5
1 mm
135 µm
1928 µm
100 µm
180 µm
C
tubing
PDMS plug
mineral oil
fluorocarbon oil
with surfactant
emulsions
D
A
B
C
D
E
F
G
H

Supplementary Figure 1 Schematics of the microfluidic devices

Blue colour denotes fluidic channels, black – channels for ground electrode, red – signal electrode and the green colour indicates auxiliary channels for insertion of optical fibres.

A) Design of the integrated device used for barcoded bead and single-cell co-encapsulation followed by fluorescence-activated droplet sorting. 1) Input channel for the barcoded compressible microgel loading. 2) Input channel for the cell loading, 3) input channel for, the lysis mix, 4, 5, 7) input channel for the fluorinated oil with admixed surfactant, 6) input channel for aqueous solution for generation of buffer droplets. 8) Outlet for negative droplets, 9) outlet for positive droplets.

B) Design of the picoinjector device, used to inject the RT enzyme mix. 1) Input channel for droplet reinjection, 2) emulsion diluting oil inlet, 3) droplet spacing oil inlet, 4) inlet for the RT mix to be picoinjected, 5) droplet exit channel.

C) Scheme of the container tip used for droplet collection and reinjection in the picoinjector device. The tip is connected to a glass syringe which enables aspiration and delivery of emulsions. The tip can be connected to a PDMS plug to close the system for incubation in the water bath.

D) Photographs presenting consecutive steps of FADS chip assembly. a**)** Mounting of the chip on the microscope stage. b) Insertion of the detection fibre. c) Insertion of the excitation fibre. d) Connecting tubing with NaCl solution and filling of salt electrodes. Please note that outlet tubings do not touch the microscope, and they are placed inside Eppendorf tubes e) Connecting tubing with lysis mix, buffer and oil/surfactant mixtures. f) Insertion of cell loading tip. g) Insertion of droplet collection tip and the negative outlet tubing. h) Insertion of the tubing with barcoded beads. Final device ready for encapsulation in a dark room required to protect UV-cleavable beads. Refers to Extended Data Figure 1A with design.

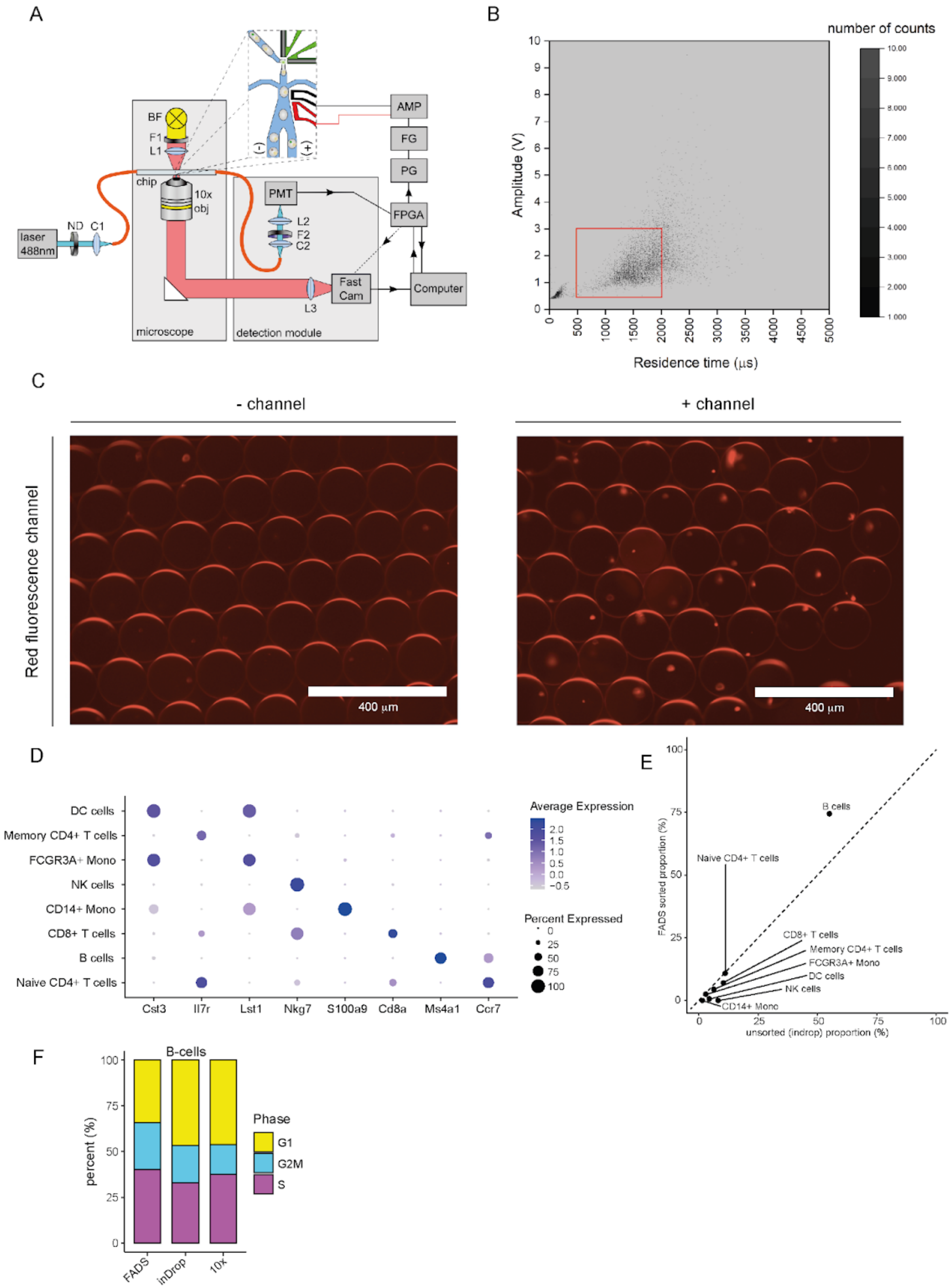
A
BF
F1
L1
chip
10x
obj
laser
488nm
ND
C1
(-)
(+)
AMP
FG
PG
PMT
FPGA
L2
F2
C2
Fast
Cam
Computer
L3
microscope
detection module
B
number of counts
Amplitude (V)
Residence time (µs)
C
- channel
+ channel
Red fluorescence channel
400 µm
400 µm
D
DC cells
Memory CD4+ T cells
FCGR3A+ Mono
NK cells
CD14+ Mono
CD8+ T cells
B cells
Naive CD4+ T cells
Cst3
Il7r
Lst1
Nkg7
S100a9
Cd8a
Ms4a1
Ccr7
Average Expression
Percent Expressed
E
B cells
Naive CD4+ T cells
CD8+ T cells
Memory CD4+ T cells
FCGR3A+ Mono
DC cells
NK cells
CD14+ Mono
FADS sorted proportion (%)
unsorted (indrop) proportion (%)
F
B-cells
percent (%)
Phase
G1
G2M
S
FADS
inDrop
10x

Supplementary Figure 2 FADS set-up and sorting

A) Scheme of the optical setup used for fluorescent activated droplet sorting for cells stained with Calcein-AM . The fluorescent light signal was recorded by the PMT coupled to a FPGA NI card and analysed by a custom written LabVIEW program in real-time. Black arrows indicate the direction of signals and triggers in the system. When a fluorescent light signal was higher than an arbitrarily set threshold, the FPGA card triggered the generation of a high voltage pulse (1 kV) by the series of electronic devices: pulse generator PG (TGP110, Thurlby Thandar Instruments) to function generator FG (TG200, Thurlby Thandar Instruments) to high voltage amplifier AMP (610E, Trek).

B) FADS sorting dataset showing the recorded fluorescent signal data points collected across an experiment. The sorted droplets in the red rectangle (intensity 0.5-3V, time 500-2000 us) represent viable HEK293T cells stained with Calcein-AM, and excludes the background empty droplets (bottom left) and droplets containing cell doublets with high fluorescence signal (right handside).

C) PBMC sorting outputs for cells stained with CD19, IgM and CD45R PE-labelled antibodies. Positive channel (+) indicates the sorting channel, negative channel (-) indicates the unsorted channel. 95.3% of droplets contain a single-cell in the + channel (n=43 droplets).

D) DotPlot for cell type-specific marker genes for the shared embedding between the 10x v2, inDrop FADS sorted for B-cells (using CD45R, CD19 and IgM-PE labelled antibodies), and the inDrop datasets generated from frozen C57BL/6 PBMCs cells.

E) Cell type proportional representation between the inDrop FADS sorted B-cells and unsorted inDrop PBMCs dataset showing an enrichment for B-cells when using FADS.

F) Cell-cycle phase inference for B-cells in the PBMC sorting experiment across datasets, showing that fractions of cells in each phase are globally equivalent across datasets.

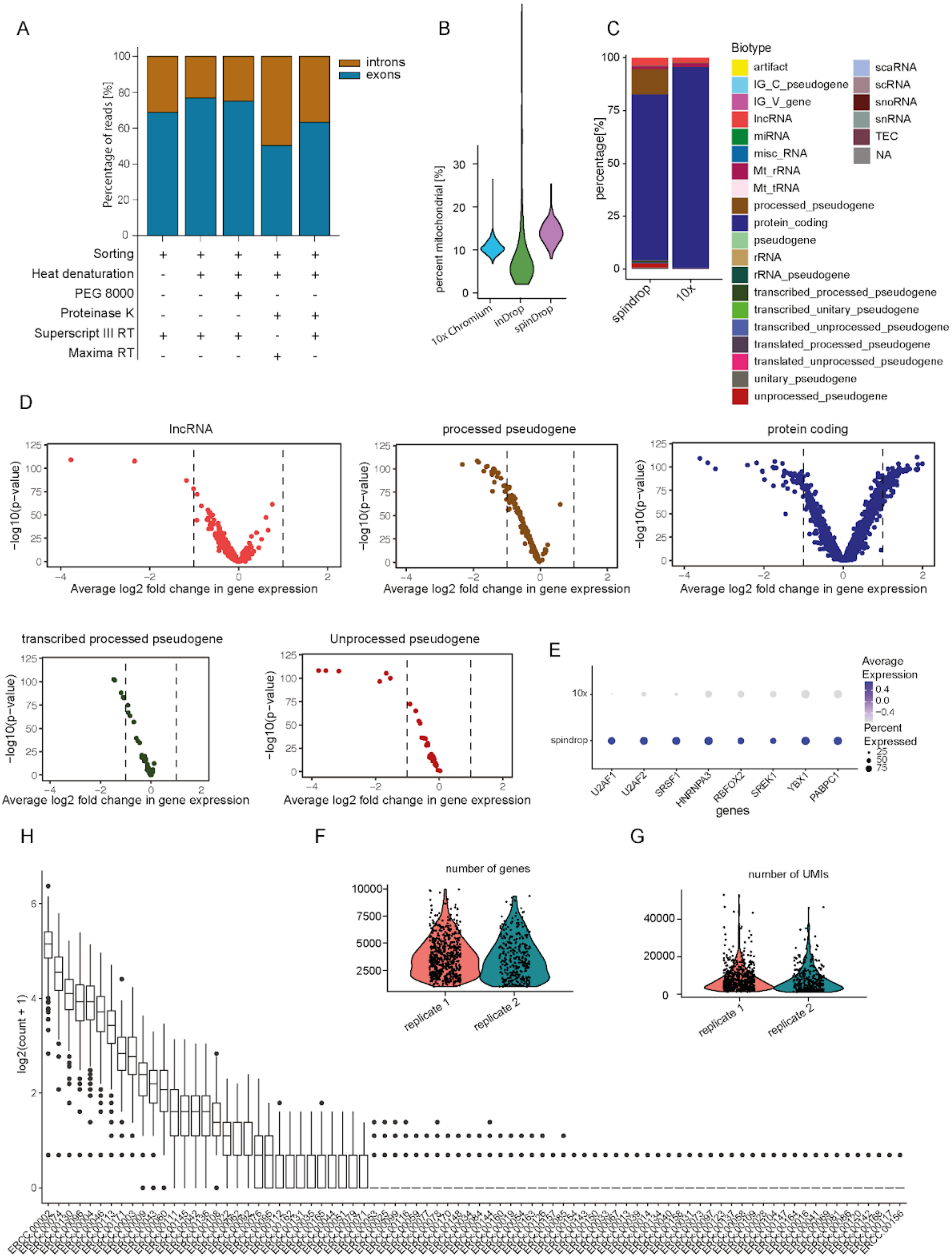
A
Percentage of reads [%]
introns
exons
Sorting
Heat denaturation
PEG 8000
Proteinase K
Superscript III RT
Maxima RT
B
percent mitochondrial [%]
10x Chromium
inDrop
spinDrop
C
percentage[%]
spindrop
10x
Biotype
artifact
IG_C_pseudogene
IG_V_gene
lncRNA
miRNA
misc_RNA
Mt_rRNA
Mt_tRNA
processed_pseudogene
protein_coding
pseudogene
rRNA
rRNA_pseudogene
transcribed_processed_pseudogene
transcribed_unitary_pseudogene
transcribed_unprocessed_pseudogene
translated_processed_pseudogene
translated_unprocessed_pseudogene
unitary_pseudogene
unprocessed_pseudogene
scaRNA
scRNA
snoRNA
snRNA
TEC
NA
D
lncRNA
processed pseudogene
protein coding
−log10(p−value)
Average log2 fold change in gene expression
transcribed processed pseudogene
Unprocessed pseudogene
E
10x
spindrop
U2AF1
U2AF2
SRSF1
HNRNPA3
RBFOX2
SREK1
YBX1
PABPC1
genes
Average Expression
Percent Expressed
H
log2(count + 1)
F
number of genes
replicate 1
replicate 2
G
number of UMIs
replicate 1
replicate 2

Supplementary Figure 3 spinDrop biotype repartition and species-mixing assay

A) Proportional representation of UMI-corrected reads mapping to intronic and exonic regions for different reaction mixtures, showing larger intronic representation with proteinase K and heat denaturation treatment.
B) Percentage of UMIs mapping to mitochondrial genes for HEK293T cells profiled using the 10x Chromium v2, inDrop and spinDrop methods.
C) RNA biotype proportional representation between 10x and spinDrop HEK293T cells, downsampled to 20,000 reads per cell. Source data are provided as a Source Data file.
D) Differential expression analysis using a Wilcoxon rank sum test between HEK293T cells processed using spinDrop (negative $\log_2$fold change values) and 10x (positive $\log_2$fold change values) for lncRNAs, processed pseudogenes, protein coding genes, transcribed processed pseudogenes and unprocessed pseudogenes.
E) Differential expression for HEK293T cells between the spinDrop and 10x datasets identifies preferential capture of splicing factors and members of the human spliceosome in the spinDrop dataset, illustrating the potential for spinDrop to uniquely uncover biological processes.
F) violin plot representing the number of genes per cell between both HEK293T replicates profiled with spinDrop.
G) violin plot representing the number of UMIs per cell between both HEK293T replicates profiled with spinDrop.
H) ERCC molecules detected across all sampled droplets from a mouse ES and human HEK293T species-mixing experiment, showcasing the ability of spinDrop to capture spike-ins at a reduced cost through exclusion of empty droplets.

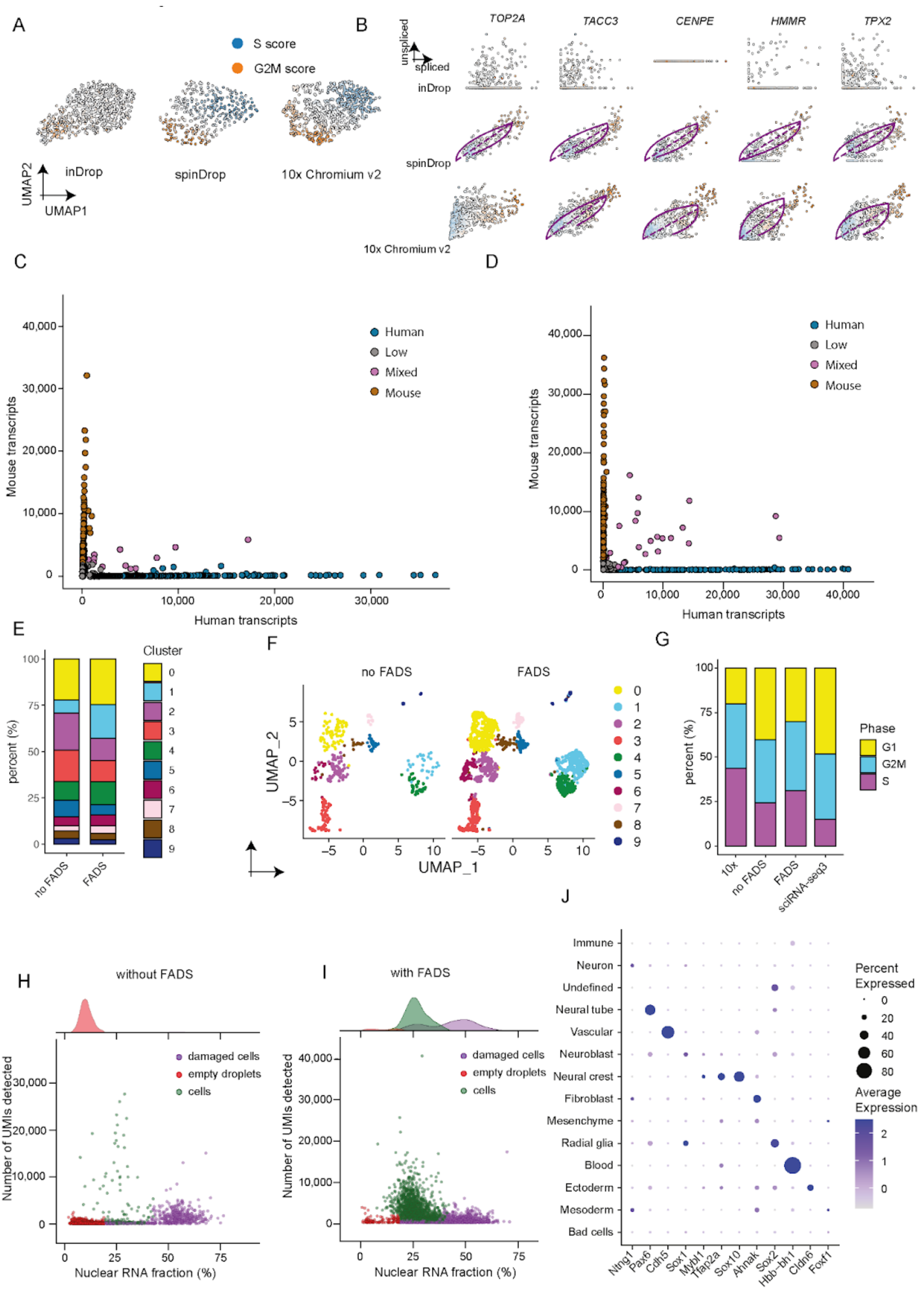

A
S score
G2M score
inDrop
spinDrop
10x Chromium v2
UMAP2
UMAP1
B
TOP2A
TACC3
CENPE
HMMR
TPX2
unspliced
spliced
inDrop
spinDrop
10x Chromium v2
C
Human
Low
Mixed
Mouse
Mouse transcripts
Human transcripts
D
Human
Low
Mixed
Mouse
Mouse transcripts
Human transcripts
E
Cluster
percent (%)
no FADS
FADS
F
no FADS
FADS
UMAP_2
UMAP_1
G
Phase
G1
G2M
S
percent (%)
10x
no FADS
FADS
sciRNA-seq3
H
without FADS
damaged cells
empty droplets
cells
Number of UMIs detected
Nuclear RNA fraction (%)
I
with FADS
damaged cells
empty droplets
cells
Number of UMIs detected
Nuclear RNA fraction (%)
J
Immune
Neuron
Undefined
Neural tube
Vascular
Neuroblast
Neural crest
Fibroblast
Mesenchyme
Radial glia
Blood
Ectoderm
Mesoderm
Bad cells
Ntng1
Pax6
Cdh5
Sox1
Mybl1
Tfap2a
Sox10
Ahnak
Sox2
Hbb−bh1
Cldn6
Foxf1
Percent Expressed
Average Expression

Supplementary Figure 4 spinDrop enables RNA spike-ins and delivers high-quality data from damaged samples.

A) UMAP dimensional reduction of the HEK293T datasets generated using inDrop, spinDrop and the 10x Chromium v2 protocols; colour gradient indicates cell cycle phase.
B) Phase plots of the top G2M cell-cycle candidates displaying dynamical expression in the spinDrop dataset. The analysis shows that increased sensitivity in the spinDrop protocol recovers cell-cycle dynamics which cannot be predicted using inDrop.
C) Species-mixing Barnyard plot using mES and human HEK293T whole cells, processed with the spinDrop protocol.
D) Species-mixing Barnyard plot using mES and human HEK293T extracted nuclei, processed with the spinDrop protocol.
E) cluster distribution for the mouse brain dataset at E10.5 with and without FADS, showing essentially similar cell-type proportions apart from fibroblasts (cluster 1).
F) UMAP representation of the datasets represented in E)
G) Cell-cycle phase inference for the mouse brain at E10.5 sorting across datasets, showing that fractions of cells in different cell-cycle phases are essentially equivalent, refuting that size distribution (hence also fluorescence) impacts the sorting process.
H) DropletQC quality metrics for the spinDrop embryo dataset without FADS sorting, showing large representation of barcodes for damaged cells and empty droplets.
I) DropletQC quality metrics for the spinDrop embryo dataset with FADS sorting, showing large representation of barcodes labelled as viable cells.
J) DotPlot representing core cell type marker expression for different cell types in the spinDrop embryonic dataset.

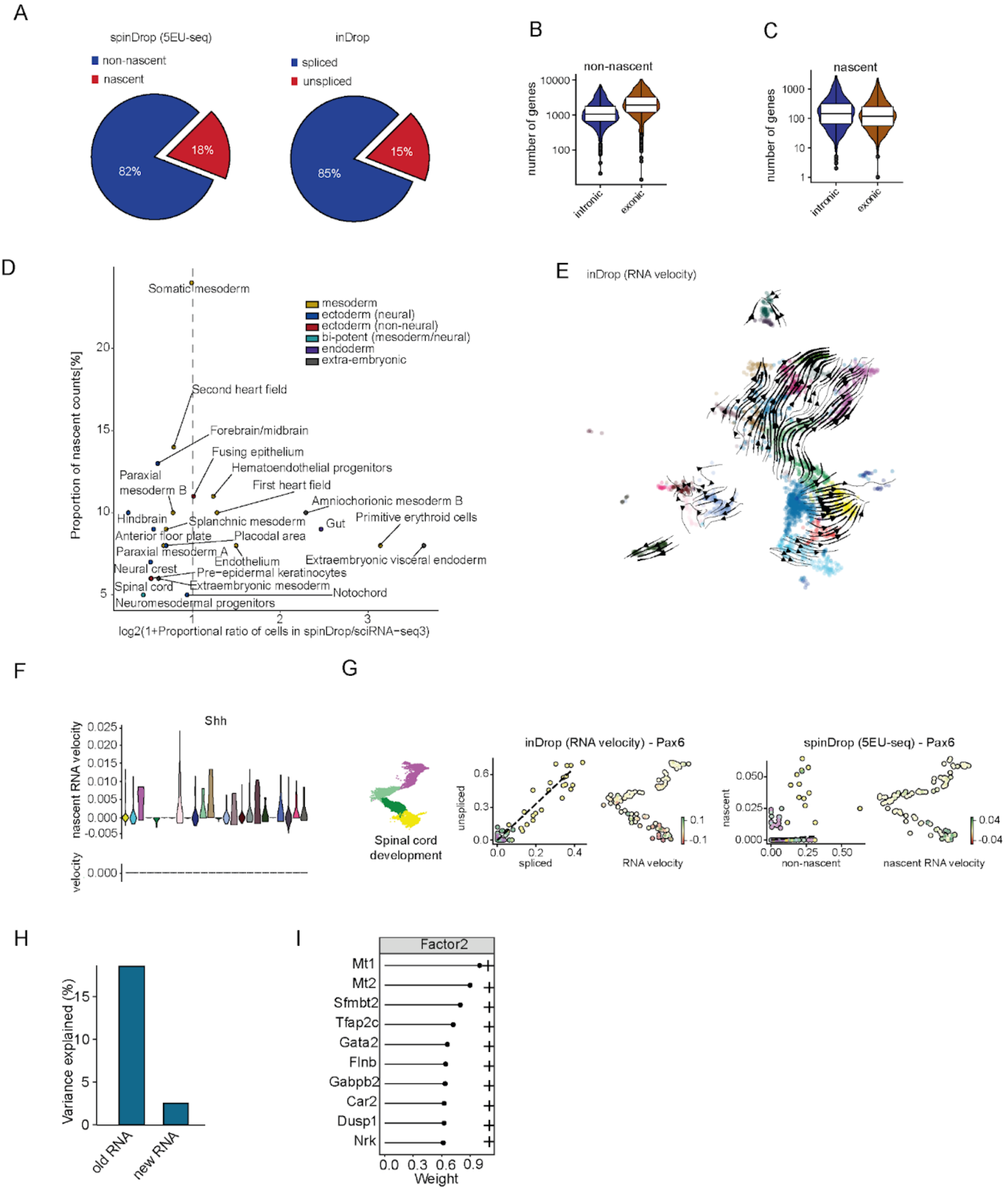
A
spinDrop (5EU-seq)
non-nascent
nascent
18%
82%
inDrop
spliced
unspliced
15%
85%
B
non-nascent
number of genes
10000
1000
100
intronic
exonic
C
nascent
number of genes
1000
100
10
1
intronic
exonic
D
Proportion of nascent counts[%]
log2(1+Proportional ratio of cells in spinDrop/sciRNA−seq3)
mesoderm
ectoderm (neural)
ectoderm (non-neural)
bi-potent (mesoderm/neural)
endoderm
extra-embryonic
Somatic mesoderm
Second heart field
Forebrain/midbrain
Fusing epithelium
Hematoendothelial progenitors
Paraxial mesoderm B
First heart field
Amniochorionic mesoderm B
Hindbrain
Splanchnic mesoderm
Gut
Primitive erythroid cells
Anterior floor plate
Placodal area
Paraxial mesoderm A
Endothelium
Extraembryonic visceral endoderm
Neural crest
Pre−epidermal keratinocytes
Spinal cord
Extraembryonic mesoderm
Notochord
Neuromesodermal progenitors
E
inDrop (RNA velocity)
F
Shh
nascent RNA velocity
velocity
G
Spinal cord development
inDrop (RNA velocity) - Pax6
unspliced
spliced
RNA velocity
spinDrop (5EU-seq) - Pax6
nascent
non-nascent
nascent RNA velocity
H
Variance explained (%)
old RNA
new RNA
I
Factor2
Mt1
Mt2
Sfmbt2
Tfap2c
Gata2
Flnb
Gabpb2
Car2
Dusp1
Nrk
Weight

Supplementary Figure 5 Nascent RNA velocity sequencing using droplet-based 5EU-seq.

A) Proportion of nascent RNA in the spinDrop 5EU-seq (left) and unspliced read in the E8.5 inDrop mouse organogenesis dataset (right).
B) Number of genes detected using reads mapping to intronic and exonic regions in the non-nascent RNA 5EU-seq dataset generated using spinDrop. Data in the box plot represent the 25%, median (centre) and 75% percentiles with minimum and maximum values.
C) Number of genes detected using reads mapping to intronic and exonic regions in the nascent RNA 5EU-seq dataset generated using spinDrop. Data in the box plot represent the 25%, median (centre) and 75% percentiles with minimum and maximum values.
D) Nascent RNA content across cell-types. The proportion of cells per cell-type with a matching barcode between nascent and non-nascent RNA generated using spinDrop is compared to the proportion per cell-type generated using an equivalent sciRNA-seq3 dataset (log2(1+n) transformed on the x-axis), in function of nascent RNA fraction (y axis). Cell-types coloured by broad tissue classes. Allantois was omitted from the analysis because of an overlap with low-complexity barcodes, affecting the purity of the cells in that cluster and nascent RNA metrics. The 5EU-seq dataset was filtered to barcodes containing reads in both the nascent and non-nascent libraries.
E) RNA velocity vectors computed using scVelo projected on the inDrop dataset of mouse organogenesis at E8.5.
F) Nascent RNA and RNA velocity plots for *Shh* across the spinDrop and inDrop datasets.
G) *Pax6* RNA velocity profiles and nascent RNA velocity profiles across the spinal cord developmental trajectory.
H) Percent variance in the datasets explained by the non-nascent (old) and nascent (new) normalised matrices identified using MOFA.
I) Top weights for factor 2 identified using MOFA showing enrichment for mitochondrial gene and core morphogenetic transcription factors. Source data are provided as a Source Data file.
