# Supplementary material for: spinDrop: a droplet microfluidic platform to maximise single-cell sequencing information content
Source: Nat Commun. 2023 Aug 8;14:4788. doi: 10.1038/s41467-023-40322-w (PMC10409775; doi:10.1038/s41467-023-40322-w)
Supplement: Supplementary file 2 — Reporting Summary [file 41467_2023_40322_MOESM2_ESM.pdf]

nature portfolio

Corresponding author(s): Florian Hollfelder
Last updated by author(s): Jun 22, 2023

Reporting Summary

Nature Portfolio wishes to improve the reproducibility of the work that we publish. This form provides structure for consistency and transparency in reporting. For further information on Nature Portfolio policies, see our Editorial Policies and the Editorial Policy Checklist.

Statistics

For all statistical analyses, confirm that the following items are present in the figure legend, table legend, main text, or Methods section.

n/a	Confirmed	
☐	☒	The exact sample size (*n*) for each experimental group/condition, given as a discrete number and unit of measurement
☐	☒	A statement on whether measurements were taken from distinct samples or whether the same sample was measured repeatedly
☐	☒	The statistical test(s) used AND whether they are one- or two-sided<br>*Only common tests should be described solely by name; describe more complex techniques in the Methods section.*
☒	☐	A description of all covariates tested
☐	☒	A description of any assumptions or corrections, such as tests of normality and adjustment for multiple comparisons
☐	☒	A full description of the statistical parameters including central tendency (e.g. means) or other basic estimates (e.g. regression coefficient) AND variation (e.g. standard deviation) or associated estimates of uncertainty (e.g. confidence intervals)
☐	☒	For null hypothesis testing, the test statistic (e.g. *F*, *t*, *r*) with confidence intervals, effect sizes, degrees of freedom and *P* value noted<br>*Give P values as exact values whenever suitable.*
☒	☐	For Bayesian analysis, information on the choice of priors and Markov chain Monte Carlo settings
☒	☐	For hierarchical and complex designs, identification of the appropriate level for tests and full reporting of outcomes
☐	☒	Estimates of effect sizes (e.g. Cohen's *d*, Pearson's *r*), indicating how they were calculated

Our web collection on statistics for biologists contains articles on many of the points above.

Software and code

Policy information about availability of computer code

Data collection	All datasets were obtained by sequencing on Illumina Nextseq 500 75 cycle high-output flow cells with the following parameters: Read1 61 cycles, Read2 8 cycles, Read3 8 cycles and Read4 14 cycles.
Data analysis	Data was inspected with FastQC v0.11.9, BCL files were converted to fastq files using the bcl2fastq script v2.19.0. The reads were then de-multiplexed per sample using Pheniqs v2.1.0 and further processed with zUMIs v2.9.1 to generate count matrices. Seurat v3 was then used for downstream analysis and integration and scVelo v0.2.5 was used for velocity analysis. DropletQC v1.0 was used to identify barcodes corresponding to alive cells. Scripts employed for these analyses can be found at https://github.com/droplet-lab/spinDrop.

For manuscripts utilizing custom algorithms or software that are central to the research but not yet described in published literature, software must be made available to editors and reviewers. We strongly encourage code deposition in a community repository (e.g. GitHub). See the Nature Portfolio guidelines for submitting code & software for further information.

Data

Policy information about availability of data

All manuscripts must include a data availability statement. This statement should provide the following information, where applicable:

- Accession codes, unique identifiers, or web links for publicly available datasets
- A description of any restrictions on data availability
- For clinical datasets or third party data, please ensure that the statement adheres to our policy

The sequencing data are available at the following accession number GSE208156 (https://www.ncbi.nlm.nih.gov/geo/query/acc.cgi?acc=GSE208156). The 1:1 3T3

and HEK293T mixture 10x Chromium v2 dataset used for benchmarking HEK293T cells is available on their website in the 'Datasets' category (1k 1:1 Mixture of Fresh Frozen Human (HEK293T) and Mouse (NIH3T3) Cells). The sciRNA-seq3 E8.5 mouse dataset was obtained from the TOME dataset (https://shendure-web.gs.washington.edu/content/members/cxqiu/public/nobackup/tome_summary_data/mm/seurat_object_E8.5b.rds); similarly to the E10.5 mouse dataset (https://shendure-web.gs.washington.edu/content/members/cxqiu/public/nobackup/tome_summary_data/mm/seurat_object_E10.5.rds) . The 10x v1 mouse brain dataset was downloaded from SRA with accession number PRJNA637987 (https://www.ncbi.nlm.nih.gov/bioproject/PRJNA637987/). The inDrop mouse organogenesis dataset at E8.5 is available on GEO with accession number GSE189425 (https://www.ncbi.nlm.nih.gov/geo/query/acc.cgi?acc=GSE189425).
The designs in Extended data Figure 1A can be found in our repository DropBase (https://openwetware.org/wiki/DropBase:Devices). Source data are provided with this paper.

Human research participants

Policy information about studies involving human research participants and Sex and Gender in Research.

Reporting on sex and gender	N/A
Population characteristics	N/A
Recruitment	N/A
Ethics oversight	N/A

Note that full information on the approval of the study protocol must also be provided in the manuscript.

Field-specific reporting

Please select the one below that is the best fit for your research. If you are not sure, read the appropriate sections before making your selection.

☒ Life sciences ☐ Behavioural & social sciences ☐ Ecological, evolutionary & environmental sciences

For a reference copy of the document with all sections, see nature.com/documents/nr-reporting-summary-flat.pdf

Life sciences study design

All studies must disclose on these points even when the disclosure is negative.

Sample size	For each sample, sample size was determined to cover each cell-type with at least ten cells per cluster to enable sufficient downstream statistical power for differential expression analysis, for example. Emulsions were collected in batches of ~1,000 cells as to keep barcode collision rates low. For proof-of-concept experiments with cultured cells with low estimated variability (HEK293T cells and mESC cells), the sample size was ~ 500 cells. For more complex datasets with multiple cell-types (PBMCs, mouse brain sequencing and 5EU-seq), the sample size was between 1,000 and 2,000 cells.
Data exclusions	Low quality cells were filtered out of the datasets where appropriate in datasets generated using alternative technologies (10x Chromium mainly) according to protocol-specific instructions, based on low gene and UMI counts and abnormal fraction of reads mapping to mitochondrial RNAs. For the 5EU-seq dataset, low-quality empty droplet cells were excluded from the dataset.
Replication	Datasets were benchmarked against existing datasets generated using 10x Chromium, inDrop and sciRNA-seq3 in order to estimate cross-method reproducibility. For spinDrop replicate analysis, two independent replicates were generated from HEK293T cells, yielding reproducible metrics across replicates.
Randomization	There was no allocation of test subjects for any experiments, thus randomization was not applicable to our study.
Blinding	For experimental setup and downstream analysis, the researchers needed to know samples, cell types and protocols. No blinding was performed. Data analyses were performed by unbiased software programs/algorithms whenever possible.

Reporting for specific materials, systems and methods

We require information from authors about some types of materials, experimental systems and methods used in many studies. Here, indicate whether each material, system or method listed is relevant to your study. If you are not sure if a list item applies to your research, read the appropriate section before selecting a response.

Materials & experimental systems

n/a	Involved in the study	
	x	Antibodies
	x	Eukaryotic cell lines
x		Palaeontology and archaeology
	x	Animals and other organisms
x		Clinical data
x		Dual use research of concern

Methods

n/a	Involved in the study	
x		ChIP-seq
x		Flow cytometry
x		MRI-based neuroimaging

Antibodies

Antibodies used	PE-anti mouse CD19 (#130-112-035), CD45R (#130-110-846) and IgM (#130-116-312) were obtained from Miltenyi Biotec.
Validation	All REAfinity antibodies sold by the manufacturer undergo rigorous validation and testing, before they obtain the validation stamp that enables shipment to the customer. The validation steps ensure: 1) high-quality antibody purification and quality-control, 2) lot-to-lot performance assessment and confirmation, 3) counter staining, knock-out of target protein, siRNA and competition assays, antibody over-expression and binding to purified antigen and cross-reactivity estimation; 4) antibody sensitivity estimation. In addition, stained cells were carefully evaluated using fluorescence microscopy post-staining to evaluate if the ratio of stained cells is in line with the expected proportions of cell-types in each sample.

Eukaryotic cell lines

Policy information about cell lines and Sex and Gender in Research

Cell line source(s)	HEK293T cells were a gift from Marc de la Roche (Department of Biochemistry, University of Cambridge). Mouse embryonic stem cells E14Tg2a wild-type were a generous gift from Prof. Austin Smith. Frozen Splenocyte from C57BL/6 mouse were ordered from Caltag medsystems (#SC-M5540-57).
Authentication	None of the cells were authenticated.
Mycoplasma contamination	Cells were not screened for Mycoplasma contamination.
Commonly misidentified lines (See ICLAC register)	No commonly misidentified strains were used in this study.

Animals and other research organisms

Policy information about studies involving animals; ARRIVE guidelines recommended for reporting animal research, and Sex and Gender in Research

Laboratory animals	CD-1 mice were obtained from the Department of Physiology, Development and Neuroscience. Mouse age ranged from 6 to 10 weeks.
Wild animals	No wild animals were used in this study.
Reporting on sex	Both male and female embryos were used for this study, none were genotyped for sex.
Field-collected samples	No field-collected samples were used in this study.
Ethics oversight	All experiments performed were under the regulation of the Animals (Scientific Procedures) Act 1986 Amendment regulations 2012 and were reviewed by the University of Cambridge Animal Welfare and Ethical review body (AWERB). Experiments were also approved by the Home Office.

Note that full information on the approval of the study protocol must also be provided in the manuscript.
