# Supplementary material for: spinDrop: a droplet microfluidic platform to maximise single-cell sequencing information content
Source: Nat Commun. 2023 Aug 8;14:4788. doi: 10.1038/s41467-023-40322-w (PMC10409775; doi:10.1038/s41467-023-40322-w)
Supplement: Supplementary file 4 — Description of Additional Supplementary Files [file 41467_2023_40322_MOESM4_ESM.pdf]

Title: Supplementary Movie 1
Description: FADS sorting in the positive channel of a droplet containing a single viable cell.

Title: Supplementary Movie 2
Description: Picoinjection of a reverse transcriptase mix into the incoming droplets.

Title: Supplementary Software 1
Description: Design files of the microfluidic designs employed in the study, including the FADS droplet generator and sorter and the picoinjector.

Title: Supplementary Data 1
Description: 1:1 dead/alive theoretical cell loading calculations

Title: Supplementary Data 2
Description: Wilcoxon rank sum two-sided differential expression test between 10x and spinDrop using HEK293T cells. Bonferroni-adjusted p-values and uncorrected p-values are provided.

Title: Supplementary Data 3
Description: Wilcoxon rank sum two-sided differential expression test between 10x and spinDrop using neuroblast cells from the mouse brain atlas. Bonferroni-adjusted p-values and uncorrected p-values are provided.

Title: Supplementary Data 4
Description: List of dynamical genes in the 5EU-seq and inDrop datasets computed using scVelo.

Title: Supplementary Data 5
Description: Fisher two-sided test computing the lineage drivers for endothelium specification for the 5EU-seq and inDrop datasets.

Title: Supplementary Data 6

Description: Cost per cell calculation to run a spinDrop experiment for the equipment, sequencing and library preparation.

Title: Supplementary Data 7

Description: Number of cells profiled throughout the study.

Title: Supplementary Data 8

Description: Photolithography protocol details for the FADS microfluidic device.

Title: Supplementary Data 9

Description: Photolithography protocol details for the picoinjector microfluidic device.

Title: Supplementary Data 10

Description: instrumentation costs and catalogue for running spinDrop
